# The behavioral phenotype of Rubinstein–Taybi syndrome: A scoping review of the literature

**DOI:** 10.1002/ajmg.a.62867

**Published:** 2022-06-21

**Authors:** Neelam Awan, Effie Pearson, Lauren Shelley, Courtney Greenhill, Joanne Tarver, Jane Waite

**Affiliations:** ^1^ School of Life Sciences and Education Staffordshire University Stoke‐on‐Trent UK; ^2^ School of Psychology College of Health and Life Sciences, Aston University Birmingham UK; ^3^ Present address: Royal Manchester Children's Hospital Manchester UK

**Keywords:** behavioral phenotype, cognition, mental health, Rubinstein–Taybi syndrome, socio‐communication

## Abstract

Rubinstein–Taybi syndrome (RTS) is a rare genetic syndrome associated with growth delay, phenotypic facial characteristics, microcephaly, developmental delay, broad thumbs, and big toes. Most research on RTS has focused on the genotype and physical phenotype; however, several studies have described behavioral, cognitive, social, and emotional characteristics, elucidating the behavioral phenotype of RTS. The reporting of this review was informed by PRISMA guidelines. A systematic search of CINAHL, Medline, and PsychINFO was carried out in March 2021 to identify group studies describing behavioral, cognitive, emotional, psychiatric, and social characteristics in RTS. The studies were quality appraised. Characteristics reported include repetitive behavior, behaviors that challenge, intellectual disability, mental health difficulties, autism characteristics, and heightened sociability. Findings were largely consistent across studies, indicating that many characteristics are likely to form part of the behavioral phenotype of RTS. However, methodological limitations, such as a lack of appropriate comparison groups and inconsistency in measurement weaken these conclusions. There is a need for multi‐disciplinary studies, combining genetic and psychological measurement expertise within single research studies. Recommendations are made for future research studies in RTS.

## 1 INTRODUCTION

### 1.1 Prevalence and genetic cause

Rubinstein–Taybi syndrome (RTS) is a multiple congenital syndrome that occurs in approximately one in 100,000 to 125,000 live births; however, genetic confirmation of diagnosis can only be obtained in approximately 65%–70% of cases (Hennekam et al., 1990; Stevens, 2019). In 1992, the first genetic anomalies for RTS were discovered in chromosome 16 including breakpoints, mutations and microdeletions (Lacombe et al., 1992). Following this, the CREBBP gene, located at 16p13.3, has been found to be affected in approximately 50%–60% of individuals (Bartsch et al., 2005; Schorry et al., 2008). A smaller number of individuals (3–10% of cases) are affected by a mutation of gene EP300 (Fergelot et al., 2016; Negri et al., 2015; Roelfsema et al., 2005; Zimmermann et al., 2007). Subsequent research literature often refers to these two etiologies of RTS as type 1 (CREBBP) and type 2 (EP300). The genetic variant leading to RTS is unknown in approximately 30% of clinically confirmed cases (Bartsch et al., 2005).

### 1.2 Clinical characteristics

Since RTS was first identified in 1957 by Michail, Matsoukas, and Theodorou, a physical, cognitive, and behavioral profile has been established. Several distinctive physical features have been identified including a small head and short stature, a characteristic facial appearance including downward‐slanting palpebral fissures, raised nasal bridge, arched eyebrows, small upper lip, micrognathia, and broad thumbs and toes (Hennekam, 2006; Rubinstein & Taybi, 1963; Schorry et al., 2008; Udwin & Dennis, 1995). A range of health difficulties are associated with the syndrome including congenital heart defects, renal system abnormalities, gastroesophageal reflux, recurrent respiratory infections, constipation, increased risk of both benign and cancerous tumors, and eye, dental, and skeletal abnormalities (Baker, 1987; Hennekam et al., 1990; Kinirons, 1983; Rubinstein, 1990; Stevens & Bhakta, 1995; Wiley et al., 2003).

Cognitive characteristics include intellectual disability (ID), ranging from mild to severe, difficulties with short term memory, delayed speech, and poor attention (Hennekam et al., 1992; Stevens et al., 1990; Waite et al., 2016). An IQ >70 has been reported some RTS individuals with a EP300 variant, although very few individuals with this genetic variant have been described (Fergelot et al., 2016). Further research has shown a wider IQ range (36–102) in individuals with the CREBBP variant using shorter non‐verbal assessment tools that rely less on language, attention, and motor skills (Ajmone et al., 2018). Behavioral characteristics associated with RTS include hyperactivity, impulsivity, and repetitive behaviors (e.g., repetitive speech and body stereotypy); some particular repetitive behaviors may be more frequent in RTS compared with other rare genetic conditions and autism spectrum disorder (ASD) (Waite et al., 2015). Age‐related changes have also been described in RTS, with reports that mood difficulties and temper tantrums increase with age (Hennekam et al., 1992). Behavioral, cognitive, and emotional characteristics associated with RTS may differ dependent the pathogenic variant and further research is needed to examine genotype–phenotype correlations.

### 1.3 Establishing the behavioral phenotype of Rubinstein–Taybi syndrome

The term behavioral phenotype was introduced by Nyhan (1972) who argued observed behaviors were integral to genetic conditions and emphasized organic etiology. Since then, more widely accepted definitions have been introduced by Dykens (1995), who conceptualizes a behavioral phenotype as the *increased likelihood* of individuals with a particular condition displaying a behavior or set of behaviors *relative to individuals* who do not have that condition (Dykens, 1995); and O'Brien (2006) who describes it as a distinctive pattern of social, linguistic, cognitive, and motor observations normally associated with a biological or genetic disorder. Cognitive and emotional characteristics are often included under the umbrella term “behavioral phenotype” despite not being directly observable, as these characteristics can be indirectly measured and have been demonstrated to influence behavior (Flint, 1996; Waite et al., 2014).

Describing the behavioral phenotype associated with a genetic syndrome is of importance to families, carers, and individuals with a genetic syndrome. For example, when supporting their child, parents request further information on topics that align with core phenotypic characteristics associated with their child's syndrome (Pearson et al., 2018). A thorough description of the behavioral phenotype of a syndrome improves understanding and helps clinicians develop targeted advice (Waite et al., 2014). Furthermore, describing behavioral phenotypes informs the development of interventions and can improve decisions about how to adapt the environment to suit a person. There are numerous examples of how behavioral phenotype research has improved practice, such as research in Cornelia de Lange syndrome (CdLS). Self‐injurious behavior is prevalent in CdLS, as it is in many rare genetic syndromes; however, in CdLS self‐injury has been specifically associated with gastro‐esophageal reflux. This knowledge has led to international clinical recommendations for the assessment of self‐injury in CdLS and subsequent treatment (Kline et al., 2018).

This scoping review aims to describe the behavioral phenotype of RTS by identifying literature that comments on the behavioral, cognitive and social characteristics of RTS. Mental health problems will be included in this review as they are often associated with cognitive, emotional, social and behavioral profiles (Waite et al., 2014). The literature will be summarized followed by an evaluation of the quality of the methodology applied in the studies for the purpose of drawing conclusions about the behavioral, cognitive, social, and psychiatric profile of RTS. Recommendations for further research will be identified.

## 2 METHOD

The reporting of this scoping review aligns with the standards of the Preferred Reporting Items for Systematic reviews and Meta‐Analyses extension for Scoping Reviews (PRISMA‐ScR) (Tricco et al., 2018).

### 2.1 Search strategy

A search of CINAHL, Medline, and PsycINFO was carried out on 3rd March 2021 and included search terms relevant to the name of the syndrome and the cognitive, behavioral, and emotional phenotype (for a full list of search terms see Table 1). Truncations (*) were used to ensure alternative word endings were included and to allow for variations in spelling. The “AND” and “OR” functions were used to combine relevant search terms, and the advanced search function was used for phenotypic characteristics.

**TABLE 1:** Search terms used for the identification of relevant articles

Syndrome search terms	“Rubinstein‐Taybi syndrome,” “Rubinstein Taybi syndrome,” “Rubinstein Taybi,” “Rubinstein‐Taybi,” “Broad Thumb Hallux” “16p13.3”
Cognitive, behavioral, and emotional phenotype search terms	[behavio* or psychiatr* or psycholog* or emotion or mood or “mental health” or social* or Autism or Autistic or “Autis* Spectrum Disorder” or ASD or Cogniti* or “executive function” or “attention deficit hyperactivity disorder” or ADHD or intelligen* or intellectual* or IQ or “mental illness” or “adaptive function” or psychosocial or affect* or hyperactiv* or impulsiv* or overactiv* or “repetitive behavio*” or aggression or aggress* or “problem behavio*” or “challenging behavio*”]

#### 2.1.1 Selection of studies

**FIGURE 1:**
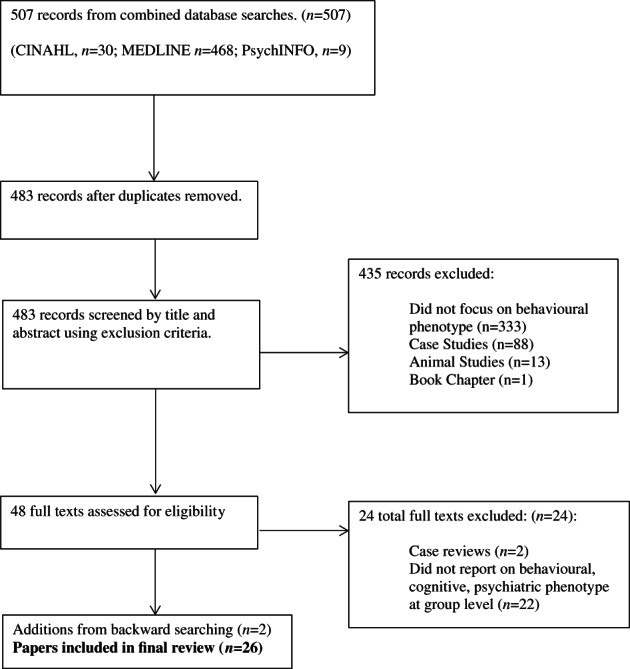
PRISMA flowchart (Page et al., 2021)

A total of 507 articles were identified. Following the removal of duplicates, 483 articles remained, and these were then screened by title and abstract (Stage 1 screening). Table 2 outlines the exclusion criteria used during the selection of studies. At Stage 1 screening, a total of 435 studies were excluded, including the following: studies where the cognitive, behavioral, emotional, psychiatric, or social phenotype of RTS, or genotype–phenotype correlations, were not the primary focus of the study (*n* = 333), case studies (*n* = 88), animal studies (*n* = 1), and book chapters (*n* = 1). The full texts of the remaining 48 studies were accessed at Stage 2, and 24 were deemed appropriate to include in the review due to containing results detailing the cognitive, behavioral, emotional, psychiatric, or social characteristics of RTS (see Figure 1). Several case series reporting on genotype–phenotype correlations were included due to (1) of the focus on of genotype–phenotype correlations in the abstract indicating the possibility of aggregated group‐level data, (2) full‐text screening identifying that these papers reported aggregated data at group level, and (3) the importance of delineating characteristics associated with EP300 and CREBBP pathogenic variants (see Table 2 for criteria). The articles and reference list of the final 24 papers were backward searched, and additional two genotype–phenotype papers were added to the review as they provided aggregated group‐level data, resulting in 26 papers.

**TABLE 2:** Exclusion criteria used in selection of papers

*Stage 1: Abstract search exclusion criteria*
Case studies, reviews/meta‐analyses, books, chapters
Case Series, unless abstract eluded to the possibility of aggregated genotype–phenotype data.
Not peer‐reviewed
Non‐human studies
Behavioral, emotional, cognitive, psychiatric, social characteristics, or genotype–phenotype correlations are not the main focus of the study.
Study of participants without RTS
Studies of mixed diagnoses if RTS is not commented on separately
*Stage 2: Abstract search exclusion criteria*
All criteria above with the addition of: Genotype–phenotype paper that did not comment on emotional, cognitive, psychiatric or social characteristics in full text

### 2.2 Data extraction and synthesis

Data extracted from each paper included demographic information, recruitment strategy, information on the genetic confirmation of the RTS sample, comparison groups, measures included, and key findings arising from the papers. These data were recorded using a table by a primary researcher. A second researcher reviewed the accuracy of data extraction for 25% of the studies. Any errors or omissions were highlighted and amended by the second reviewer. The results in this review are presented in a narrative method and the themes were derived from discussion between researchers and behavioral phenotype literature.

### 2.3 Quality review

The literature was appraised using a quality framework adapted from a meta‐analysis on the prevalence of ASD in rare genetic syndromes (Richards et al., 2015). The adapted framework focuses on five domains tailored to genetic syndrome research that reflect key threats to internal and external validity: sample identification (e.g., via syndrome support groups), level of confirmation diagnosis (e.g., clinical diagnosis, genetic testing), inclusion of comparison groups, properties of behavioral/psychological measures, and properties of cognitive assessments (see Table 3 for full criteria). Each domain was scored from 1 (poor) to 4 (excellent) producing a total score for each paper. If a paper did not include behavioral assessment or cognitive assessment, the respective domain was not scored. The total score was then divided by the highest possible score (20 for 5 domains, 16 for 4 domains) to produce a final score ranging from 0 (lowest possible score) to 1 (highest possible score). All scores were reported to two decimal places (see Table 4). A second researcher also completed quality ratings using the chosen quality framework for 25% of the papers to confirm the reliability of the ratings. An excellent level of inter‐rater reliability (96.67%) was achieved.

**TABLE 3:** Appraisal criteria adapted from Richards et al. (2015)

	1‐Poor	2‐Adequate	3‐Good	4‐Excellent
Sample identification	Not specified/reported	Single, restricted, or non‐random sample (e.g., from a specialist clinic or previous research study)	Multiple restricted or non‐random sample (e.g., multiregional specialist clinics)	Random or total population sample
Confirmation of syndrome^a^	Unreported/ unconfirmed Clinical diagnosis only suspected	Clinical diagnosis by general clinician (e.g., general practitioner, pediatrician) Diagnosis by application of broad diagnostic criteria	Clinical diagnosis by “expert” clinician (e.g., clinical geneticist)	Genetic confirmation Confirmation is made clinically **and** genetic testing was carried out during the study
Behavioral/ psychological assessment	Descriptions of behavior based on non‐standardized informant report, or review of clinical information, or too little information to categorize	Standardized informant report measure (e.g., the RBQ, ADI‐R) or clinical judgment based on DSM or ICD criteria	Standardized behavioral or observational assessment (e.g., neuropsychiatric evaluation, ADOS)	Consensus drawn from multiple assessments, including one or more standardized behavioral or observational assessment
Cognitive assessment	Description or estimation of cognitive ability based on non‐standardized informant report, or review of clinical information, or too little information to categorize	Standardized informant report measure (e.g., VABS)	Standardized behavioral or observational assessment (e.g., neuropsychiatric evaluation, BSID)	Consensus drawn from multiple assessments, including one or more standardized behavioral or observational assessment
Comparison groups	Unreported No control group Does not compare to standardized scores	Compares with standardized scores in general population Compares with a historical control group (e.g., control group from previous studies)	Compares with standardized scores in comparable population (e.g., intellectual disability) Compares with a concurrent control group	Compares with a concurrent control group that is matched by age and gender, as well as other features pertinent to the research question

Abbreviations: Repetitive Behavior Questionnaire (RBQ; Moss et al., 2009); Autism Diagnostic Interview ‐ Revised (ADI‐R; Lord et al., 1994); Diagnostic and Statistical Manual of Mental Disorders (DSM); International Classification of Disease (ICD); Autism Diagnostic Observation Schedule (ADOS; Lord et al., 2000); Vineland Adaptive Behavior Scale II (VABS‐II; Sparrow et al., 2005); Bayley Scales of Infant and Toddler Development (BSID; Bayley, 1969).

^a^ Studies can only be classified into a category if all of the participants were tested using the outlined method. For instance, if only 50% of participants were genetically tested as part of the study, the study cannot receive a score of 4 and will receive a score of 3.

**TABLE 4:** Summary of all studies and quality scoring

Authors (year)	Study aims	Recruitment strategy	RTS sample *N* age (age range) *genetic mechanism*	Non‐RTS comparison group *N* (age range)	Measures to characterize behavioral phenotype	Findings: Behavioral, cognitive, emotional profile	Quality score
Ajmone et al. (2018)	To evaluate the genotype–phenotype correlations in individuals with RTS	Unspecified	*N* = 23 Mean age: 7.6 years (18 months–20 years) *20 = CREBBP* *1 = EP300* *2 = non conclusive*	None	The Griffiths Scales (Griffiths, 1986); Leiter‐R; CBCL; SCQ Lifetime *Not all participants completed the direct assessment measures*	No ID: (4/16; 25%); Mild ID: (3/16; 18.7%); Moderate ID: (9/16; 56.2%); No evaluation (severe ID; 2/23; 0.9%) No DD: (3/14; 21.4%); Mild DD: (4/14; 28.6%); Moderate DD: (5/14; 35.7%); Severe DD: (2/14 14.2%) Fluid reasoning higher than IQ scores Clinical Score of ASD: (37%) Children aged 1.5–5 years: Internalizing problems: (41.7%); Attention problems: (41.7%) Children aged 6–18 years: “Total competence problems”: (100%); Social problems: (89%)	0.60
Boer et al. (1999)	To describe the developmental and behavioral aspects of RTS.	Syndrome support group	*N* = 44 Mean age: 12 years (3–51 years) 31 children (3–16 years) 13 adults (17–51 years) *Genetic mechanism unconfirmed*	None	SSBP‐PQ	Repetitive speech: children (57%); adults (84.6%) Repetitive movements: children (77.4%); adults (38.5%) Usual Routines: children (53.3%); adults (69.2%) Self‐injury: children (45.2%); adults (53.8%) Verbal abuse: children (86.2%); adults (84.6%) “Too friendly with strangers”: children (77.3%); adults (33.3%) Serious temper outbursts (at least weekly) (29.5%)	0.38
Crawford et al. (2020)	To investigate social anxiety and social motivation in individuals with CdLS, FXS & RTS	Participant database at research institution	*N* = 20 Mean age: 25.52 years *Confirmed diagnosis from professional; genetic mechanism unreported*	CdLS Mean age: 22.62 years FXS Mean age: 23.68 years DS Mean age: 23.67 years	BPVS‐II; VABS‐II; SCQ; Social Anxiety and Motivation Rating Scale	In the RTS group, heightened levels of behavior indicative of social anxiety was observed across a range of social situations Participants with FXS and RTS demonstrated higher levels of social anxiety than those with DS Participants with RTS did not differ from those with DS for indicators of social motivation	0.75
Crawford et al. (2015)	To further the understanding of whether the documented differences in social behavior in RTS and CdLS are subcortically or cognitively mediated.	Participant database at research institution & syndrome support group	*N* = 17 Mean age: 17.33 (4–37 years) *Confirmed diagnosis from professional; genetic mechanism unreported*	CdLS: 15 (6–33 years)	Eye‐Tracking Task; SCQ; VABS‐II	No significant differences between the RTS group and the CdLS group on the amount of time spent looking at the face stimuli Both groups spent more time looking at disgust faces compared with neutral faces but not a higher amount of time looking at happy faces compared with disgust faces Conclusion: differences in sociability between RTS and CdLS are unlikely to be subcortically mediated	0.75
Crawford et al. (2016)	To determine if individuals with CdLS, FXS and RTS demonstrate attentional differences to social vs. non‐social stimuli	Participant database at research institution and syndrome support group	*N* = 19 Mean age: 20.94 years *Confirmed diagnosis from professional; genetic mechanism unreported*	CdLS: 14 Mean age: 18.21 years FXS: 15 Mean age: 24.21 years	Eye‐Tracking Task; VABS‐II; SCQ; SCAS‐P	Individuals with RTS exhibited increased attentional maintenance of attention towards socially salient compared with less salient social stimuli. They also showed increased attention prioritization to social stimuli compared with individuals with CdLS.	0.75
Crawford et al. (2017)	To enhance understanding of anxiety in individuals with RTS, FXS and CdLS by investigating anxiety at symptom level.	Syndrome Support Group	*N* = 27 Mean age: 23.55 years *Confirmed diagnosis from professional; genetic mechanism unreported*	CdLS:13 FXS:19 Typically developing children: 261 Typically developing children with anxiety: 484	VABS‐II; SCQ; SCAS‐P	High levels of social phobia in the RTS group compared with the typically developing control group Lower levels of panic/agoraphobia and OCD in the RTS group compared with typically developing control group No differences between the RTS group and the typically developing participants with anxiety for panic/agoraphobia and OCD 43.75% of RTS group met the cut off for ASD	0.60
Ellis et al. (2020)	To characterize the profiles of sociability in individuals with CdLS, FXS, and RTS by comparing.	Participant database at research institution & syndrome support groups	*N* = 25 Mean age:15.22 (2–59 years) *Confirmed diagnosis from professional; genetic mechanism unreported*	CdLS: 36 (2–50 years) FXS: 36 (2–46 years)	VABS‐II; MSEL; BAS3; CSRS	The RTS group did not display behaviors indicative of sociability more frequently or of greater quality than individuals with CdLS and FXS Lower levels of positive emotional affect and reduced quality of eye contact were associated with higher scores on a measure of autism in individuals with RTS Individuals with RTS did not show any associations between age and different components of sociability.	0.80
Fergelot et al. (2016)	To evaluate the genotype–phenotype correlations in individuals with RTS	Five laboratories in Europe that offer EP300 testing	*N* = 360 Mean age: unreported *52: EP300 Mutation* *308: CREBBP Mutation*	No non‐RTS comparison groups EP300 and CREBBP groups compared	A questionnaire (non‐specified) was used to obtain information from clinicians	94% of the EP300 mutation group had an intellectual disability (Severe = 7%, Moderate = 31%, Mild = 62%) 99% of the CREBBP mutation group had an intellectual disability (Severe = 36%, Moderate = 48%, Mild = 14%) 25% of the EP300 mutation group had autism/ autistiform behavior 49% of the CREBBP mutation group had autism/ autistiform behavior	0.56
Galéra et al. (2009)	To determine whether behavioral features in children with RTS, differ from those found in children with intellectual disability of heterogeneous etiology.	Syndrome association and a university department of medical genetics	39 Mean age: 8.4 years (4.3–15.8 years) *CREBBP in 49% cases +* *Clinical diagnosis to confirm ‘classic case’*	Typically developing children:39 (4.4–15.5 years)	CBCL; CBSQ	Lower levels of anxiety in the RTS group compared with the control group Poorer attention and concentration in the RTS group than the control group RTS group displayed significantly more motor stereotypies including “flaps arms and hands when excited,” “makes odd or fast movements with fingers or hands,” and “extremely pleased by certain movements/keeps doing them” than the control group Higher levels of sociability in the RTS group compared with the control group No differences for mood or temper disturbances between RTS group and the control group	0.81
Giacobbe et al. (2016)	To describe the electroclinical phenotype of patients with RTS and correlate with genetic, cognitive and neuroradiological features	Syndrome association and referral through a single clinic	23 Mean age: 7 years 2 months (18 months–20 years) *20 = CREBBP* *1 = EP300* *1 = confirmed clinically* *1 = unconfirmed*	None	Leiter‐R; The Griffiths Scales	Reported results at group level No ID: (17%) Mild ID: (13%) Moderate ID: (39%) Developmental Disability (DD) Normal developmental: (21%) Mild DD: (28%) Moderate DD: (36%) Severe DD: (14%)	0.69
Gotts & Liemohn, 1977	To document the behavioral characteristics of children with RTS.	Unreported	3 (7–10 years) *Diagnosed based on clinical characteristics*	Children with intellectual disability of heterogeneous etiology: 15	Leary and Coffey (1955) Behavioral Checklist	The matched control group showed significantly lower levels of the following compared with the RTS group Anxiety symptoms Short attention span difficulties Overreaction to stimulation/being highly excitable Accepting social contact Residual anger	0.44
Hennekam et al. (1992)	To document the psychological examinations of individuals with RTS	Recruitment through Dutch journals for professionals and parents, lectures to parents and professionals, through national institutions, TV, ID and non ID schools Estimated that half population of Netherlands recruited.	40 (2.7–60.3 years) *Diagnosed based on clinical characteristics*	For analysis of social competency and temperament only, compared RTS with existing data from “a group of persons with mental retardation”	WISC‐R; WPPSI; Stutsmans Intelligence Test (Cattell, 1940); BSID; Achenbach Behavior Checklist (Achenbach, 1979)	RTS IQ mean = 35.6; range = 25–79 There is a sharp decline in IQ as age increases RTS group displayed higher levels of social competence when compared with control group data Common behavior problems occurred in over 25% of the RTS group 41% of the RTS group were reported to have “temper tantrums.”	0.50
Levitas and Reid (1998)	To report the psychiatric evaluation of individuals with RTS	Six state centres referring to a single university site for genetic evaluation	*N* = 13 Mean age: 39.7 (24–51 years) *Clinical diagnosis and blood specimen: No deletions found. No analysis for point mutations*.	None	Psychiatric Assessment	IQ ranged from mild to severe No association between IQ and psychiatric diagnosis Mood disorders prevalence (8/13) Tic/OCD prevalence (4/13) Schizophrenia, Generalized Anxiety Disorder and panic were not observed	0.65
López et al. (2018)	To report the molecular and clinical characteristics of individuals with RTS arising from EP300 mutations	Drawn from a cohort of 72 individuals with suspected RTS after being negative in a CREBBP study	*N* = 8 (3 months–21 years) *8 = Mutations in EP300*	None	None reported	Psychomotor delay was observed in 4 cases Mild ID: (3/7) Moderate ID: (4/7) Severe ID: (1/8) Language delay was observed in 3 cases Autism/autism‐like was reported in 3 cases	0.45
Moss et al. (2016)	To examine the nature and developmental trajectory of sociability in AS, CdLS, DS, FXS and RTS.	RTS family support group	88 (4–49 years) *Confirmed diagnosis from professional. Genetic mechanism unreported*	AS: 66 (aged 4–48 years) CdLS: 98 (4–43 years) FXS: 142 (9–49 years) DS: 117 (4–62 years)	SQUID; Wessex Scale (Kushlick et al., 1973); SCQ	Higher levels of sociability in RTS, Angelman syndrome and Down syndrome compared with CdLS, fragile X syndrome and ASD in various social contexts Individuals with RTS and Angelman syndrome shared a similar level of sociability except for “initiating interaction” where the Angelman syndrome group scoring significantly higher High levels of “extreme socialibility” in RTS group during familiar and unfamiliar social situations	0.55
Negri et al. (2015)	To identify the clinical and molecular characterization of RSTS patients carrying novel EP300 inactivating mutations	From cohort of >200 cases of whom 188 entered CREBBP study. A subset had EP300 molecular analysis Recruitment from the Italian patients' and families association	*N* = 6 (3–25 years) 6 = *Mutations in EP300*	None	Clinical evaluation (unspecified)	’Pronounced anxiety’ in 3/6 patients Psychomotor delay: 5/6 Borderline/mild ID: 2/6 Mild/moderate: 1/6 Moderate ID: (2/6)	0.50
Negri et al. (2016)	To identify the clinical and molecular characterization of RSTS patients carrying novel EP300 inactivating mutations	Identified from cohort of 22 CREBBP negative patients Italian patients' and families association	*N* = 6 (7–23 years) *6 = Mutations in EP300*	None	Clinical evaluation (unspecified)	Psychomotor delay: 3/6 Language delay: 3/6 Moderate ID: 3/6 Mild ID: 3/6 ‘Autism like’ (*N* = 1); attention deficit (*N* = 1); poor interaction with classmates and medical examiners (*N* = 2); sleep disturbances (*N* = 1); aggressiveness with psychosis to severe anxiety (*N* = 1)”	0.50
Pérez‐Grijalba et al. (2019)	To evaluate the genotype–phenotype correlations in individuals with RTS	Referred by patient's medical center	*N* = 39 (2 months–42 years) *39 = CREBBP positive patients*	None	Structured questionnaire led by a clinical specialist	Above 85% of the sample reported psychomotor and language delays 14 patients reported ‘behavioral’ problems (anxiety, autism) Intellectual disability, ranging from mild to severe, was reported for all probands	0.45
Schorry et al. (2008)	To evaluate the genotype–phenotype correlations in individuals with RTS.	Recruited via two international family conferences (> 200 attendees)	*N* = 93 *52 CREBBP‐positive patients* 41 patients with absent or synonymous mutations	No non‐RTS group *RTS groups compared*	Medical records reviewed by 2 physicians including clinical geneticist or developmental pediatrician Developmental and school performance data (not all had formal cognitive testing) Autism features were assessed via physician interview (non‐standardized)	Self‐injurious behavior (6.5%) Aggressive behavior (10.8%) Self‐stimulatory or repetitive behaviors seen in 31% of total group IQ <50: (44.3%) IQ 50–75: (53.2%) IQ >75: (2.5%) Trend towards more autistic features and lower IQ in those with large deletions No significant differences across groups for autistic characteristics, attentional problems, hyperactivity, self‐injurious or aggressive behaviors	0.50
Spena et al. (2015)	To evaluate the genotype–phenotype correlations in individuals with RTS.	Italian reference research centre collates patient information from clinical geneticists from diverse Italian and foreign centres.	*N* = 46 *46 = CREBBP point mutations*	None	Clinical evaluation from referring clinical specialist. Clinical Questionnaire (unspecified)	Reported results at group level Mild/borderline ID: (26.7%) Moderate ID: (43.3%) Severe ID: (26.7%)	0.50
Stevens et al. (1990)	To document the development and behavior of individuals with RTS living in institutions.	National RTS syndrome support group	50 (1–26.5 years) Confirmed diagnosis by clinical geneticist and clinical data consistent with diagnosis. No molecular analysis reported	None	Parental Questionnaire (no further details provided) ICAP ‐ maladaptive behavior section only	IQ mean = 51 (range = 30–79) Short attention span: (90%) Sensitivity to sound: (46%) “Unusual behaviors” including self‐stimulatory behaviors: (65%) Moderate‐ serious maladaptive behaviors: (10%)	0.44
Stevens et al. (2011)	To document the medical issues, education, independence and behavior problems in adults with RTS.	Recruited through a research database, syndrome support group and RTS list serve	*N* = 61 Mean age: 28.5 years (18–67 years) *Diagnosed by clinical geneticist or other specialist. No molecular studies of CREBBP or EP300*	None	140 item caregiver questionnaire covering medical problems, education, independence and behavior	Attention span: (72%) Distractibility: (70%) Impulsivity (56%) Disruptive actions: (29%) Psychiatric diagnosis: the majority of which were OCD, anxiety, or depression: (31%) Self‐injury: (32%) Autistic type behaviors: ‐ needing a strict routine: (62%); intolerance of noise/crowds: (62%); difficulty with change in the environment: (62%); and self‐stimulation behaviors: (61%).	0.45
Waite et al. (2015)	To compare the profile of repetitive behaviors in RTS, ASD, DS and FXS; to explore the association between repetitive behavior and ASD phenomenology across groups; to explore associations between repetitive behavior and degree of disability across groups	Syndrome support group database	87 (aged 4–59 years) *Confirmed diagnosis from professional. Genetic mechanism unreported*	ASD: 228 (4–45 years) Fragile X syndrome:196 (aged 6–47 years) Down syndrome: 132 (4–62 years)	Wessex Scale; SCQ; RBQ	RTS group scored higher on stereotyped behavior and compulsive behavior compared with Down syndrome group RTS had significantly higher levels of repetitive speech than Down Syndrome No differences were found for restricted preferences and insistence on sameness for RTS compared with other syndromes RTS group showed heightened levels of stereotypy, hoarding, preference to routine, repetitive questions and phrases compared with Down syndrome group RTS had lower levels of restricted conversation, repetitive phrase and echolalia than ASD and fragile X syndrome, lower levels of adherence to routine and hand stereotypy than fragile X syndrome RTS had heightened scores on body stereotypy compared with Down syndrome. Lower levels of restricted conversation and repetitive phrases relative compared with ASD group and fragile X syndrome.	0.60
Waite et al. (2016)	To explore the cross‐sectional developmental trajectories of working memory domains in RTS.	Syndrome support group and university database	*N* = 21 (aged 6–37 years) *Previously confirmed diagnosis from professional; genetic mechanism unreported*	Typically developing children: 89 (3–7 years)	MSEL; WASI‐II; VABS‐II; Verbal Animal Scan (Bull et al., 2004); Corsi Blocks (Pickering et al., 1998); Scrambled Boxes (Diamond, 1990)	RTS working memory varied depending on the aspect of working memory measured The typically developing control group consistently outperform RTS group with higher mental age performed better than on verbal and visuo‐spatial working memory Differences in the trajectory of working memory‐ RTS trajectory remains flat in contrast to a positive slope in the control group	0.75
Wincent et al. (2016)	To describe clinical presentations in a cohort of Swedish patients with RTS	Single specialist clinic	*N* = 17 (1–32 years) *11 = CREBBP pathogenic mutations* *2 = EP300 mutations* *3 = CREBBP intronic variations of unknown significance*	None	Clinical data (no further details provided)	“Behavioral” problems (anxiety and/or aggression): 8/17 Autism: 5/17 Variable degree of intellectual disability	0.45
Yagihashi et al. (2012)	To compare behavioral difficulties in children and adults with RTS	RTS syndrome support group	*N* = 63 Median = 13 years (1–38 years) *Molecular tests had been performed in 20 of 56 participants. Nine had identified molecular abnormalities (EP300 or CREBBP)*	None	CBCL	Older individuals (>14 years) with RTS scored higher on anxiety, depression, nervous highly strung or tense, attention difficulties, too fearful/anxious compared with younger group (≤13 years) with RTS Older individuals with RTS showed significantly more aggressive behavior than the younger individuals (*p* = 0.036)	0.56

*Note*: Instrument abbreviations: The Leiter International Performance Scale Revised (Leiter‐R; Roid & Miller, 1997); Child Behavior Checklist (CBCL; Achenbach, 2001); Social Communication Questionnaire (SCQ; Rutter et al., 2003); The Study of Behavioral Phenotypes Postal Questionnaire (SSBP‐PQ; O'Brien, 1995); The British Picture Vocabulary Scale II (BPVS‐II; Dunn et al., 1997); Vineland Adaptive Behavior Scale II (VABS‐II; Sparrow et al., 2005); Mullen Scales of Early Learning (MSEL; Mullen, 1995); British Ability Scales 3rd Ed. (BAS3; Elliott & Smith, 2011); The Child Sociability Rating Scale (CSRS; Moss et al., 2013); Children's Social Behavior Questionnaire (CBSQ; Luteijn et al., 1998); Wechsler Intelligence Scale for Children Revised (WISC‐R; Wechsler, 1973); Wechsler Preschool & Primary Scale of Intelligence (WPPSI; Wechsler, 1989); Bayley Scales of Infant and Toddler Development (BSID; Bayley, 1969); Sociability Questionnaire for People with Intellectual Disability (SQUID; Moss et al., 2016); The Inventory for Client & Agency Planning (ICAP; Bruininks et al., 1986); Repetitive Behavior Questionnaire (RBQ; Moss et al., 2009); Wechsler's Abbreviated Scale of Intelligence (WASI‐II; Wechsler, 2011).

## 3 RESULTS

### 3.1 Summary of participants and study quality

The results obtained from the papers and the quality ratings are summarized in Table 4. Overall, 1238 participants were included across all studies, including 515 and 76 with confirmed CREBBP and EP300 genetic variants, respectively. However, it is possible that there was some overlap in samples across studies due to the rarity of syndrome and shared recruitment routes. Sample sizes were typically small within the individual studies, with considerable variation in sample sizes across the studies (range: 3–360). Most studies recruited participants from regional or national syndrome support groups.

The mean quality rating for all studies was 0.57 (range: 0.38–0.81). The mean quality ratings for each quality criterion were as follows: sample identification = 2.35, confirmation of RTS diagnosis = 2.85, use of comparison groups = 2.04, properties of behavioral/psychological measures = 1.96, and properties of cognitive assessments = 2.25. The main factors that affected the quality ratings of the studies were lack of methodological information or the use of non‐standardized measures for assessing behavioral, cognitive, and emotional characteristics. The use of non‐standardized or unspecified measures was common in studies where the methodology for confirming genetic diagnosis was excellent (e.g., Fergelot et al., 2016; Negri et al., 2016). Conversely, studies that were rated highly on the quality of the methodology for assessing behavior and associated characteristics typically did not score as highly on diagnostic confirmation due to not conducting genetic testing as part of the study (e.g., Ellis et al., 2020; Waite et al., 2015). There was also a lack of comparison groups in 14 of 26 studies; however, when comparison groups were included, half were high‐quality matched concurrent samples (6/12 studies).

### 3.2 Behavioral characteristics

Ten of the twenty‐six studies presented findings focusing on self‐stimulatory or repetitive behaviors, aggressive behavior, and self‐injurious behavior.

#### 3.2.1 Self‐stimulatory/repetitive behavior

Self‐stimulatory/repetitive behaviors are consistently reported as a feature of the RTS behavioral phenotype across six studies. Stevens et al. (1990) reported that 65% of their sample of children with RTS (*N* = 50) displayed “unusual behaviors,” which are reported as being primarily self‐stimulatory in nature, including rocking, spinning, and hand flapping. In a later study (*N* = 44), which covered a larger age range, repetitive speech was reported in 57% of children and 84.6% of adults; however, repetitive movements appeared to occur in fewer adults (38.5%) compared with children (77.4%) (Boer et al., 1999), as did adherence to strong routines. Neither study compared these findings to typically developing (TD) individuals, individuals with other rare genetic syndromes or ID of heterogeneous etiology.

Studies that included comparison groups have confirmed higher levels of self‐stimulatory/repetitive behaviors in RTS. When comparing children with RTS to a comparison group of TD children matched for developmental ability and chronological age, Galéra et al. (2009) found that children with RTS (*N* = 39) scored significantly higher on the items: “flaps arms/hands when excited”; “makes odd/fast movements with fingers/hands”; and “pleased by movements/keeps doing them.” When matched for ability to children with ID of heterogenous etiology, it has also been found that children (*N* = 3) with RTS displayed significantly more self‐stimulatory behaviors compared with the children without RTS, although the sample size in this study was small (Gotts & Liemohn, 1977).

Comparisons between RTS and other neurodevelopmental conditions were carried out by Waite et al. (2015) who described a specific profile of repetitive behavior in children and adults with RTS (*N* = 87) in comparison with Down syndrome (DS), fragile X syndrome (FXS), and ASD. Findings indicated stereotyped behavior and compulsive behavior occurred more frequently in individuals with RTS compared with individuals with DS but did not differ from FXS or ASD. By examining the types of repetitive behaviors displayed between the groups, at fine‐grained level of description, an uneven profile of repetitive behavior was identified that would not have been apparent if composite repetitive behavior scores were used. When total group analyses were conducted, the RTS group displaying more frequent hand, object and body stereotypy, hoarding, adherence to routines, repetitive phrases and repetitive questioning compared with the DS group. However, RTS had lower levels of restricted conversation, repetitive phrases and echolalia compared with FXS and ASD. The RTS group also showed lower levels of adherence to routine and hand stereotypy than individuals with FXS and lower levels of cleaning than in ASD. However, when matched for age, adaptive ability and verbal ability many of these differences were no longer statistically significant, although the RTS group still showed body stereotypy significantly more frequent than the DS group.

#### 3.2.2 Challenging behavior

Nine studies commented on challenging behavior including aggressive behaviors and self‐injurious behaviors. The prevalence of challenging behaviors varies across the studies with *common behavior problems* reported to occur in 25% (*N* = 40) of individuals with RTS when assessed using the Achenbach Behavior Checklist (Hennekam et al., 1992), and 10% (*N* = 50) of *maladaptive behaviors* reported to be moderate to severe (Stevens et al., 1990).

#### 3.2.3 Aggressive behaviors

Boer et al. (1999) reported high levels of “verbal abuse” in both children and adults with RTS (86.2% and 84.6% respectively; *N* = 44), whereas other studies report less prevalent aggressive behavior (10.8%; *N* = 46) (Schorry et al., 2008). The psychometric properties of the measures used to obtain the estimates in these latter two studies is unclear, and this may have impacted results. In contrast, a study of 63 children and adults with RTS using a standardized measure, the Child Behavior Checklist, reported age‐related differences with older individuals (>13 years) displaying significantly more aggressive behavior than younger individuals (Yagihashi et al., 2012). Most studies examining aggressive behaviors have been conducted with Type‐1 (CREBBP) RTS or a group of RTS participants of unspecified genetic mechanism. One study reported on aggressive behavior in six individuals with the rarer EP300 inactivating mutations (Negri et al., 2016); one participant was reported as showing “aggressiveness”; however, as there was no comparison group, it cannot be concluded from this study whether aggression is a behavioral characteristic that is associated with EP300 inactivating mutations.

#### 3.2.4 Temper outbursts

“Residual anger” has been noted as a feature of RTS when matched to a heterogenous ID group (Gotts & Liemohn, 1977). However, the number of people with RTS in this study was small (*N* = 3). Hennekam et al. (1992) reported 41% of their sample displayed temper outbursts and Boer et al. (1999) noted that 29.5% of their sample (*N* = 44) experienced “serious temper outbursts” at least weekly. In contrast, Galéra et al. (2009) found no significant difference between the RTS children and the typically developing children for “temper tantrums or hot temper”; however, this study did not contain adults, so it does not rule out age related increases in temper outbursts.

#### 3.2.5 Self‐injurious behaviors

Only three studies commented specifically on self‐injurious behavior and the prevalence estimates varied across the studies, ranging from 6.5% to 53.8% (Boer et al., 1999; Schorry et al., 2008; Stevens et al., 2011). Boer et al. (1999) reported a lower prevalence of self‐injurious behavior in children with RTS (45.2%) compared with adults with RTS (58.3%); however, Stevens et al. (2011) reported a lower prevalence of self‐injurious behavior (32%) in their sample of adults with RTS, which may be due to the use of different measures. These findings differed from Schorry et al. (2008) who reported that only 6.5% of their sample of individuals with RTS displayed self‐injurious behaviors; however, the age range of the sample is unknown, and the estimate of self‐injurious behavior was produced by examining developmental and school performance data. None of the studies reported the topography of self‐injurious behavior, and information on the severity and duration of these behaviors was absent.

All studies that have reported on aggressive behavior, temper outbursts and self‐injurious behavior have used different methods for data collection, none of which included direct observations of challenging behavior. Several studies did not report detailed information regarding the measures or method used to identify challenging behavior (e.g. Negri et al., 2016; Schorry et al., 2008; Stevens et al., 2011). Although overall the findings point towards the presence of challenging behaviors, it is not possible ascertain a profile of these behaviors without operationalized definitions of challenging behaviors and complete information on the methods used to identify them.

### 3.3 Cognitive characteristics

Seventeen studies commented on the cognitive characteristics associated with RTS, including seven that noted attentional difficulties, within their results sections (see Table 4); however, there was variability in the extent of cognitive impairment reported across studies. For example, Schorry et al. (2008) reported that 44.3% of their sample had an IQ below 50, 53.2% with an IQ between 50 and 75 and 2.5% with an IQ above 75. Another study reported a mean IQ of 35.6 (range 25–79) and a sharp decline in IQ as age increased (Hennekam et al., 1992). Ajmone et al. (2018) reported IQ and fluid reasoning scores in a group of genetically confirmed (predominantly CREBBP mutations) aged 18 months to 20 years using a standardized tool, the Griffiths Scales and the Leiter International Performance Scales—Revised. This study confirmed previous IQ estimates, which placed most individuals with RTS in the moderate ID range and indicated that fluid reasoning scores were generally higher than IQ and general quotient of development scores. Of note, is the largest study to date comparing individuals with RTS caused by differing genetic mechanisms (EP300 & CREBBP mutations), indicating that those with EP300 mutations typically have mild ID (62% of sample with EP300), whereas CREBBP mutations are typically associated with moderate to severe ID (48% and 36% of the sample respectively) (Fergelot et al., 2016). However, despite this, it is unclear whether standardized assessment measures of IQ were applied to assess degree of ID as this study used a questionnaire (non‐specified) to obtain clinical information.

One study focused on a specific domain of cognitive function and reported impairments in verbal and visuo‐spatial working memory across most age groups in people with RTS compared with typically developing children (Waite et al., 2016). There were no significant differences between the RTS group and comparison group on a visuo‐spatial working memory task at the youngest developmental age of measurement (3 years old); however, the typically developing group's cross‐sectional trajectory had a positive slope with age, whereas this remained flat for the RTS group, suggesting a particular difficulty in visuo‐spatial working memory difficulty in RTS.

### 3.4 Emotional and psychiatric characteristics

Thirteen studies discussed psychiatric or emotional difficulties in individuals with RTS (see Table 4). Levitas and Reid (1998) completed a psychiatric assessment and reported on the characteristics of 13 adults with RTS. It was identified that 8/13 of the sample had a ‘mood disorder’ and 4/13 were identified as having tics or OCD. A further study with a larger sample reported that 31% of adults with RTS had received a psychiatric diagnosis, mostly OCD, anxiety or depression (Stevens et al., 2011). In studies using standardized questionnaire measures, “internalising behavioural difficulties” were reported in 41.7% of RTS participants <20 years based on the Child Behavior Checklist (Ajmone et al., 2018). Age‐related differences were also reported in one study, with older individuals with RTS (>14 years) displaying higher levels of anxiety, depression, nervousness and fearfulness compared with younger individuals (≤13 years) with RTS (Yagihashi et al., 2012). Anxiety has been reported in individuals with RTS carrying EP300 inactivating mutations as well as those with CREBBP mutations (Negri et al., 2015; Pérez‐Grijalba et al., 2019).

Studies that compared emotional and psychiatric characteristics in RTS to individuals without RTS have shown mixed results. Two studies compared anxiety in individuals with RTS and typically developing individuals yet produced contrasting results. Crawford et al. (2017) reported significantly lower levels of social phobia in individuals with RTS, and significantly higher levels of panic/agoraphobia and OCD in comparison with typically developing normative data, however, it was noted that an OCD diagnosis in RTS should be applied cautiously given that repetitive behavior in the syndrome may be misattributed as a symptom of OCD. The scores of participants with RTS did not differ from data from children diagnosed with panic/agoraphobia, however, they were significantly lower than children diagnosed with OCD. Galéra et al. (2009) found significantly lower levels of anxiety in children with RTS compared with a comparison group of typically developing children. It is important to note that Crawford et al. (2017) had a broader age range of participants with RTS including some adults; Galéra et al. (2009) only included children with RTS, which might explain the different findings. Furthermore, the questionnaire Galéra et al. (2009) applied provides a total anxiety score and does not break anxiety down by anxiety‐type as in Crawford et al.’s (2017) study, so the differences may reflect measurement differences.

Studies comparing rates of psychiatric diagnoses in individuals with RTS to developmental disorder groups are sparse. When matched for ID, individuals with ID of heterogeneous etiology had significantly lower levels of anxiety symptoms compared with three children with RTS (Gotts & Liemohn, 1977). Cross syndrome comparisons have shown that individuals with RTS demonstrated higher levels of behaviors indicative of social anxiety across a range of social situations with both familiar and unfamiliar adults (Crawford et al., 2020).

### 3.5 Social characteristics

#### 3.5.1 Autism spectrum characteristics

Eighteen studies reported findings related to social characteristics including difficulties with social skills, social anxiety (see emotional and psychiatric section), and ASD. Stevens et al. (2011) reported behaviors pertaining to autism including requiring strict routines (62%), difficulty with tolerating noises and crowds (62%) difficulty with tolerating unexpected change (62%) and self‐stimulatory behaviors (61%); however, the authors reported that only 19% of adults with RTS were diagnosed with autism. Two other studies reported similar results with 37% and 43.75% of individuals with RTS meeting the cut off for ASD (Ajmone et al., 2018; Crawford et al., 2017) using an ASD screening tool, the Social Communication Questionnaire (SCQ; Rutter et al., 2003). Waite et al. (2015) reported that individuals with RTS on average had a moderate score on the SCQ; although, it was also reported that scores on the SCQ were likely elevated due to repetitive behavior in RTS rather than social‐communication difficulties, and that characteristics associated with autism may be dissociated in RTS. Two studies included in this review reported on autism in small groups of individuals carrying novel EP300 variants. Autism and ‘autism like’ behaviors were reported in 3/8 and 1/6 participants in these studies respectively (López et al., 2018; Negri et al., 2016). However, the absence of standardized measures or autism and small sample sizes means these findings should be interpreted with caution.

#### 3.5.2 Sociability and social interest

Social skills were examined in 12 studies, in which heightened levels of sociability or enhanced social skills were reported in 6 studies. Individuals with RTS showed higher levels of social competence compared with TD children; social contact and interest were found to be significantly higher in the RTS group (Galéra et al., 2009; Hennekam et al., 1992). These findings are consistent across most studies measuring social characteristics, with over‐friendliness reported in 77.3% of children (Boer et al., 1999) and that the RTS group “accepts social contacts readily” and significantly more than the matched comparison group (Gotts & Liemohn, 1977). Cross syndrome comparisons also showed heightened levels of sociability in individuals with RTS compared with individuals with Cornelia de Lange syndrome (CdLS), FXS and ASD (Moss et al., 2016). However, Crawford et al. (2020) findings differed. This was the first study to use observational measures of social behavior in RTS, rather than parental report measures, and showed that social interest in individuals with RTS did not differ from a comparison group matched for receptive language and adaptive behavior abilities. They suggested that social motivation may be developmentally typical.

An additional two studies aimed to understand the varied profiles of sociability observed in rare genetic syndromes. Crawford et al. (2015) explored whether the social impairment observed in CdLS and the heightened sociability observed in RTS are subcortically or cognitively mediated through the use of a face scanning task. No significant differences were observed between the two syndromes indicating that the contrasts in sociability between the two syndromes are unlikely to be subcortically mediated. However, a further study conducted by Crawford and colleagues using a similar eye tracking procedure revealed that individuals with RTS exhibited increased attention towards socially salient stimuli compared with less salient social stimuli compared with individuals with CdLS.

## 4 DISCUSSION

The results confirm that several behavioral, cognitive, social and psychiatric characteristics appear to be present in individuals with RTS, including repetitive behavior, challenging behavior, ID, heightened sociability, mood disorders, and anxiety. A key limitation is the heterogeneity of assessment methods used across the studies to measure these areas. With some studies providing minimal information about the measures used and some using non‐standardized measures, it has highlighted the need for a more robust and uniform methodology using direct and indirect tools. A further limitation that made it difficult to interpret the findings is the heterogeneity in the age of the participants across the studies, with some studies including only children and others including adults in their sample. Furthermore, some studies were dated, and some had small samples, which made identifying specific phenotype–genotype correlations very difficult. Moreover, the lack of longitudinal studies does not allow for a natural trajectory of the behavioral phenotype of RTS to be established. Although similar characteristics are often reported across studies, the lack of contrast groups in over half of the studies limits the conclusions that can be drawn regarding whether these characteristics are more likely to be displayed in someone with RTS relative to someone who does not have RTS.

### 4.1 Behavioral characteristics

The results showed variability in the prevalence estimates for repetitive movements (31% and 77.4%) (Boer et al., 1999; Galéra et al., 2009). This may partly be explained by the different tools used across the studies, as they vary in the way in which they identify and measure repetitive movements. The Study of Behavioral Phenotypes Postal Questionnaire (SSBP‐PQ; O'Brien, 1995) was used by Boer et al. (1999) and asks the respondent to indicate the presence of repetitive movements by selecting a “yes” or “no” response. This tool does not consider different types of repetitive movements displayed, unlike the Child Behavior Checklist (CBCL; Achenbach, 1991) used by Ajmone et al. (2018), Galéra et al. (2009) and Yagihashi et al. (2012) and the Repetitive Behavior Questionnaire (RBQ; Moss et al., 2009) used by Waite et al. (2015). The CBCL and the RBQ, therefore, allow for a more detailed understanding of the types of repetitive movements observed in individuals with RTS. The RBQ also measures the frequency of repetitive behaviors enhancing our understanding further.

Prevalence estimates for challenging behavior also varied across the studies and this may be explained by the lack of a clear definition of challenging behavior. Without a shared understanding of behaviors that are deemed to be challenging, it is difficult to measure the presence of those behaviors in individuals with RTS. Emerson (1995) described challenging behavior as “behaviours of such an intensity, frequency or duration that the physical safety of the person or others is likely to be placed in serious jeopardy.” Research studies that have focused on the epidemiology of challenging behavior identified specific behaviors that are considered to fall within the category of challenging behavior, including aggression, self‐injurious behavior and property destruction (Borthwick‐Duffy, 1994; Kiernan & Qureshi, 1993; Qureshi, 1994; Qureshi & Alborz, 1992). Some of the studies included in this review measured aggression and self‐injurious behavior, however other studies did not. For example Hennekam et al. (1992) reported that common behavior problems occurred in 25% of individuals with RTS; however, the tool employed in this study (Achenbach Behavior Checklist; Achenbach & Edelbrock, 1983) did not measure aggression, self‐injurious behavior or destruction of property. The types of behaviors captured by this tool include “wets bed,” “thumb‐sucking,” “picks nose,” and “temper tantrums.” Although these behaviors can be of concern, they do not necessarily fall within the definition of challenging behavior.

The studies in this review focused solely on documenting the prevalence of challenging behavior. None of the studies documented the etiology; however, this is particularly important in enhancing our understanding of the factors associated with challenging behavior in individuals with RTS. Challenging behaviors have been shown to serve as a communicative function in individuals with ID (Durand & Merges, 2001; Mirenda, 1997; Richman et al., 2001) suggesting that this is an attempt by the individual to communicate something, such as needing help, requesting access to an object or activity, or communicating dislike for something (Bopp et al., 2004; Carr et al., 2002; Horner, 2000; Kincaid et al., 2002). Exploring the functions of challenging behavior in individuals with RTS including the role of communication is imperative in allowing appropriate interventions and support to be offered.

### 4.2 Cognitive characteristics

The findings across the studies show that most individuals with RTS fall within the moderate ID range (Ajmone et al., 2018; Hennekam et al., 1992; Schorry et al., 2008); however, there was variability in the extent of cognitive impairment reported across studies. For example, Schorry et al. (2008) reported that 44.3% of their sample had an IQ below 50, 53.2% with an IQ between 50 and 75 and 2.5% with an IQ above 75. Another study reported a mean IQ of 35.6 (range 25–79) and a sharp decline in IQ as age increased (Hennekam et al., 1992).

Ajmone et al. (2018) reported IQ and fluid reasoning scores in a group of genetically confirmed (predominantly CREBBP mutations) aged 18 months to 20 years using a standardized tool, the Griffiths Scales and the Leiter International Performance Scales – Revised. This study confirmed previous IQ estimates, which placed most individuals with RTS in the moderate ID range and indicated that fluid reasoning scores were generally higher than IQ and general quotient of development scores. Of note, is the largest study to date comparing individuals with RTS caused by differing genetic mechanisms (EP300 & CREBBP mutations), indicating that those with EP300 mutations typically have mild ID (62% of sample with EP300), whereas CREBBP mutations are typically associated with moderate to severe ID (48% and 36% of the sample respectively) (Fergelot et al., 2016). However, despite this, it is unclear whether standardized assessment measures of IQ were applied to assess degree of ID as this study used a questionnaire (non‐specified) to obtain clinical information.

Most studies that included individuals with EP300 pathological variants had small sample sizes, apart from Fergelot et al. (2016), so the ability to compare characteristics between those with EP300 to CREBBP variants is limited. The findings on EP300 are also inconsistent across studies and are based on unstandardised/non‐specified assessments; however, there is some evidence indicating the EP300 variant may be associated with less severe ID and lower rates of autism relative to the CREBBP variant, and that individuals with the EP300 variant may also experience anxiety (Negri et al., 2015).

These findings indicate the importance of conducting genetic testing to confirm an RTS diagnosis; however, a number of the studies confirmed diagnosis through the presence clinical characteristics or by participants reporting that a diagnosis had previously been confirmed by a clinical geneticist or pediatrician (e.g., Moss et al., 2016; Stevens et al., 1990; Waite et al., 2016). While genetic testing is the gold standard within research studies, it is possible that the practicalities of conducting these tests within behavioral research settings is a barrier to the inclusion of these tests. In addition, the genetic mechanism leading to RTS cannot be identified in all individuals and, therefore, clinical features are still essential for the confirmation of the presence of RTS (Stevens, 2019). Interestingly, there appears to be a dissociation between the quality of genetic testing in studies and the quality of the assessments of behavior, emotion and cognition, with studies that have the highest quality ratings for syndrome confirmation (i.e. via genetic testing during the study), often reporting the use of unstandardised measures for behavioral profiling. There are examples of studies that are rated highly on both criteria (e.g. Galéra et al., 2009 **)** but these studies are less common. This most likely reflects a difference in expertise of those leading the studies (e.g., clinical geneticist led or psychologist led) and highlights the potential for increasing quality of further RTS behavioral phenotype research via collaborative working that brings together multidisciplinary expertise.

### 4.3 Emotional and psychiatric characteristics

The prevalence estimates for psychiatric difficulties in individuals with RTS is also quite variable (31%–61%). All the estimates across the studies are higher than that reported for the general population (29.2%; Steel et al., 2014), suggesting that individuals with RTS are at higher risk of developing mental health difficulties. However, it is important to consider the challenges in identifying mental health difficulties in individuals with ID. Individuals with ID may have difficulty providing verbal accounts of their emotional state, meaning traditional methods of assessment (e.g., clinical interview) may not be possible and the lack of validated diagnostic tools means that mental health difficulties may be under‐reported in RTS (Costello & Bouras, 2006; Moss et al., 1997). Conversely, several studies drew particular attention to the presence of anxiety disorders and specifically to a heightened prevalence of OCD; however, given that OCD is conceptualized by the presence of obsessive, intrusive thoughts and compulsions, often described as repetitive behaviors or rituals (American Psychiatric Association & DSM‐5 Task Force, 2013), it is possible that OCD is over‐reported in RTS due to the presence of repetitive/stereotyped behaviors. Without controlling for repetitive behaviors or assessing for the presence of obsessive/intrusive thought patterns, conclusions regarding mental health difficulties in RTS should be treated with caution.

The findings presented by Yagihashi et al. (2012) points towards age‐related differences in the psychiatric profile of RTS; however, it was not possible to establish a clear trajectory of mental health difficulties. Depression and anxiety were not reported separately adding to the challenges in understanding the mental health profile of individuals with RTS. Moreover, the chosen measure (CBCL) is not validated for use with individuals over the age of 18 years, which again highlights the need for selecting more appropriate measures to identify behavioral, cognitive and emotional characteristics in individuals with ID, particularly as a proportion of people with RTS have severe ID. There are few measures available for detecting mental health difficulties in those with severe ID, however a small number exist (Flynn et al., 2017).

### 4.4 Social characteristics

The results showed a high prevalence of ASD characteristics in individuals with RTS. These characteristics include restricted preferences, sensitivity to noise, difficulties with unexpected change and self‐stimulatory behaviors (Stevens et al., 2011). These findings are not unexpected as research has shown higher rates of ASD in rare genetic conditions compared the general population (Richards et al., 2015); however, it is important to recognize that none of the studies used comprehensive observational assessments to identify ASD, such as the Autism Diagnostic Observation Schedule (ADOS; Lord et al., 2000); therefore, we cannot say with certainty whether ASD is more prevalent in RTS. Instead, all the studies in this review used informant questionnaires to identify the presence of characteristics associated with ASD. Some of the ASD measures employed may not be validated for the identification of autism characteristics and should therefore be interpreted with caution; for example, Stevens et al. (2011) used a parental questionnaire; however, they did not elaborate on whether this was a standardized questionnaire designed to assess behavioral difficulties and ASD traits in rare genetic syndromes. Three studies (Crawford et al., 2015; Crawford et al., 2017; Waite et al., 2015) used the SCQ (Rutter et al., 2003), which is a well‐validated tool that identifies ASD characteristics in individuals with ID. The measure has high concurrent validity with the ADOS, as the total score on the SCQ is strongly related to the total score on the ADOS (Berument et al., 1999; Lord et al., 1994), therefore making it a more suitable tool to be used to assess ASD in individuals with RTS.

According to the DSM‐V (American Psychiatric Association, 2013) and the ICD‐11 (World Health Organization, 2018), autism is classified by the presence of two core features which include deficits in social interaction and communication and the presence of restrictive and repetitive patterns of behavior. The heightened or persevered social functioning that is reported in RTS appears contradictory to the findings reporting a high prevalence of ASD in RTS. High levels of repetitive behavior and seemingly preserved social functioning may suggest a dissociation of behaviors across the ASD dyad of impairments in individuals with RTS (Waite et al., 2015). Similar findings have been noted in other rare genetic syndromes, such as FXS and CdLS (Hall et al., 2010; Moss et al., 2012). For example, individuals with FXS demonstrated significantly fewer impairments across social and communicative behaviors compared with individuals with ASD, yet many individuals with FXS still meet the cut‐off for ASD using the SCQ (Hall et al., 2010). More detailed descriptions of sociability in FXS have found that although individuals with the syndrome display shyness, social anxiety and gaze avoidance, emotion sensitivity and willingness to interact may also be preserved (Cornish et al., 2007; Hall et al., 2006; Turk & Graham, 1997). Research has also shown heightened levels of ASD in individuals with CdLS based on the total ADOS score; however, domain and item specific analysis indicate individuals with CdLS show more eye contact and gestures, and less repetitive behavior and stereotyped speech than the ASD group (Moss et al., 2012). These findings, along with reports of prolonged eye gaze and heightened social anxiety in CdLS (Collis et al., 2006; Goodban, 1993), suggest that the profile of social impairments in CdLS may be different to that observed in ASD. With regard to RTS, recent research conducted following this review has indicated the benefit of examining autism characteristics and social characteristics at this level of fine‐grained description indicating nuanced differences to those observed in ASD (Adrien et al., 2021; Ellis et al., 2021; Taupiac et al., 2021).

Delineation of the profile of ASD in rare genetic syndromes clearly demonstrates how subtle differences in phenomenology can be obscured when the presence or absence of ASD is estimated solely from clinical cutoff scores. The use of questionnaires to assess ASD may have inflated prevalence estimates in RTS due to the high frequency of repetitive behavior in the syndrome. Many individuals with RTS may have met the cut off for ASD due to the presence of repetitive behavior alone.

### 4.5 Clinical implications

The findings from across the studies indicate that repetitive behavior and behaviors that challenge are likely to be specific features of RTS, thus highlighting the need for appropriate support for individuals who display these behaviors. There are no intervention studies for challenging behavior in individuals with RTS, however, there are effective interventions and clinical guidance available for behaviors that challenge in ID populations (National Institute for Health and Care Excellence, 2018). Challenging behavior has been found to be more likely in individuals who have an increased need of assistance and those who have restricted receptive and expressive communication (Emerson et al., 2001; Emerson & Bromley, 1995), so supporting the development of communication from an early age and providing increased mobility support, may help toward preventing and managing behaviors that challenge.

The use of augmentative and alternative communication (AAC) strategies including aided modalities such as PECS (Bondy & Frost, 1994) and unaided modalities such as Makaton (Walker, 1987) have been recommended for use with individuals with ID (Beukelman & Mirenda, 2013). Both unaided and aided modalities of AAC have been successfully taught to individuals with ID and severe communication difficulties (Kagohara et al., 2013; Lancioni et al., 2013; Schwartz & Nye, 2006; Sutherland et al., 2010; Wendt, 2009). Early input from speech and language therapy for individuals with RTS would therefore be very beneficial in supporting the development of communication and subsequently reducing behaviors that challenge. This will have a positive impact on the quality of life of those with RTS.

Interventions for repetitive behaviors may not be necessary unless the behavior is having a significant impact on quality of life. However, if adherence to routines becomes problematic some interventions that have been developed for other conditions may be appropriate (e.g. Bull et al., 2017). Finally, several studies have suggested that anxiety may occur in RTS. There are very few validated interventions for anxiety in people with severe to profound ID (Vereenooghe et al., 2018), however, behavioral strategies for anxiety may be able to be adapted for this group. In those with mild to moderate ID, behavioral strategies or adapted CBT may be appropriate (Hatton, 2002; Jahoda et al., 2017). There is guidance available on supporting individuals with learning disabilities who are experiencing anxiety using low‐intensity CBT (Dagnan et al., 2015). Some of the adaptations suggested for individuals with learning disabilities may be appropriate for individuals with RTS, including adjusting the length of the therapy session; providing support when filling in outcome measures; using easy read resources; focusing on behavioral aspects of an intervention; and finally considering inviting carers/family members to the session if the individual feels this would be beneficial (Dagnan et al., 2015).

## 5 LIMITATIONS OF THIS REVIEW

Although this was a systematic search, it is possible that some publications were missed if they were not listed in the identified databases. Furthermore, due to initially screening papers based on title and abstract, it is also possible that some papers commented on the behavioral phenotype of RTS in the full text but were screened out. Despite this, this review provides a useful overview of the status of the RTS literature, particularly regarding methodological issues that may preclude accurate identification of syndrome characteristics.

## 6 CONCLUSION

Research on RTS to date has made some progress in describing the behavioral phenotype of RTS. This review has highlighted the need for further research to replicate findings, to address the inconsistencies across studies and the lack of comparison groups. The varying methodology used to measure the behavioral phenotype of RTS has drawn attention to the importance of using standardized assessment tools that are appropriate for individuals with rare genetic conditions. It may be useful to create a standard criterion of instruments that are suitable for use to improve the overall quality of the research and to allow for a clearer comparison of the research findings. A thorough understanding the behavioral, cognitive, and emotional characteristics of RTS will allow for appropriate interventions to be developed and trialed to ensure that evidence‐based support is developed to help those with the condition and their families.

## AUTHOR CONTRIBUTIONS

Neelam Awan, Joanne Tarver, and Jane Waite conceptualized the study. Joanne Tarver and Jane Waite provided supervision. Neelam Awan conducted the literature screening, reviewed the extracted studies at title, abstract and full‐text level and assessed study eligibility. Jane Waite, Neelam Awan, and Effie Pearson reached consensus on final included studies. Neelam Awan, Lauren Shelley, Effie Pearson, and Jane Waite assessed study quality. Neelam Awan and Jane Waite performed data charting and analysis. Effie Pearson and Courtney Greenhill reviewed data charting and analysis for accuracy. All authors contributed to interpretation of findings and to review and revision of the manuscript. All authors approved the final manuscript prior to submission.

## CONFLICT OF INTEREST

The authors of this manuscript have no conflict of interest to declare.

## Data Availability

The data that support the findings of this study are available from the corresponding author upon reasonable request.
